# Vascular Invasion and Survival in Extremity Leiomyosarcoma: An Exploratory Single-Center Cohort Study from the Oxford Sarcoma Service

**DOI:** 10.3390/cancers18193212

**Published:** 2026-10-05

**Authors:** Seva N. S. Gill, Tanjot S. Kurala, Serkan Bayram, Jennifer M. Brown, Muhammad Riaz, Muhammad A. Siddiqi, Thomas D. A. Cosker

**Affiliations:** 1 Nuffield Department of Surgical Sciences, University of Oxford, Oxford OX1 2JD, UK; thomas.cosker@ouh.nhs.uk; 2 Oxford Sarcoma Service, Oxford University Hospitals, Oxford OX3 9DU, UK; jennifer.brown@ouh.nhs.uk (J.M.B.); muhammad.riaz1@ouh.nhs.uk (M.R.); ather.siddiqi@ouh.nhs.uk (M.A.S.); 3 Academic Department of Trauma and Orthopaedics, School of Medicine, University of Leeds, Leeds LS2 9JT, UK; tanjot.singh@nhs.net; 4 Department of Orthopedics and Traumatology, Istanbul University, 34452 Istanbul, Turkey; serkan.bayram@istanbul.edu.tr; 5 Department of Cellular Pathology, Oxford University Hospitals, Oxford OX3 9DU, UK

**Keywords:** leiomyosarcoma, vascular invasion, survival, extremity

## Abstract

Background/Objectives: Extremity leiomyosarcoma is an uncommon soft tissue sarcoma with a more favorable prognosis than other anatomical subtypes. Risk stratification currently rests on tumor grade, size, and metastatic status. Vascular invasion is an established predictor of metastasis in mixed soft tissue sarcoma cohorts but has seldom been formally examined in extremity leiomyosarcoma. We assessed its prognostic value in a single-center cohort. Methods: We retrospectively studied 62 adults who underwent resection of primary localized extremity leiomyosarcoma at a tertiary sarcoma center between January 2015 and February 2025. Vascular invasion was reported contemporaneously at original diagnostic reporting by specialist sarcoma histopathologists. Overall (OS), disease-specific (DSS), local recurrence-free (LRFS), and metastasis-free (MFS) survival were estimated by the Kaplan–Meier method and compared using log-rank tests. Death from another cause was treated as a competing event for disease-specific death and distant metastasis in a sensitivity analysis. Multivariable Cox models were refitted using Firth’s penalized likelihood, with discrimination corrected for optimism by bootstrapping. Results: Median follow-up was 59.5 months. Five-year OS, DSS, and MFS were 71.0%, 80.2%, and 74.6%. Vascular invasion was reported in 11 of 62 patients (17.7%) and was associated with inferior OS (*p* = 0.002), DSS (*p* < 0.001), and MFS (*p* < 0.001), but not LRFS (*p* = 0.631). Adjusted for grade, it was associated with inferior OS (HR 3.89, 95% CI 1.35–11.26), DSS (HR 5.48, 95% CI 1.46–20.58), and MFS (HR 5.63, 95% CI 1.69–18.80). The association persisted on separate adjustment for tumor size, depth, and neoadjuvant radiotherapy. Conclusions: Vascular invasion was associated with inferior survival in localized extremity leiomyosarcoma. With 17 deaths and 14 metastatic events, these are exploratory estimates, but they support documenting vascular invasion explicitly, as present or absent, in routine histopathological reporting so that its role can be evaluated at scale.

## Simple Summary

Leiomyosarcoma is an uncommon smooth muscle cancer. When it arises in an arm or leg, it generally behaves less aggressively than at other sites, but predicting which patients will do badly remains difficult. Vascular invasion—tumor cells growing inside blood vessels—can be seen when the tumor is examined under a microscope, but it is currently an optional item in pathology reports and has rarely been studied in limb leiomyosarcoma. We reviewed 62 patients treated at our specialist center over ten years. Vascular invasion was found in one in six tumors and was associated with a fourfold increase in the risk of death and a fivefold increase in the risk of the cancer spreading. Because the study is small, these findings should be treated as preliminary. They argue that vascular invasion should be recorded explicitly in every limb sarcoma report so that its value can be tested in larger studies.

## 1. Introduction

Soft tissue sarcomas are rare mesenchymal malignancies, and their management is complicated by a substantial risk of recurrence and limited systemic treatment options [1]. Histological subtype is a primary determinant of therapeutic response and long-term oncological outcome, with each subtype characterized by distinct molecular biology, treatment pathways, and surveillance requirements [2]. Leiomyosarcoma is defined by smooth muscle differentiation, accounts for approximately 10 to 20% of soft tissue sarcomas, and may arise wherever smooth muscle is present, including the walls of blood vessels [3].

Leiomyosarcoma of the extremities has been characterized in population-based and registry series [4,5], and carries a more favorable prognosis than uterine, retroperitoneal, or major-vessel disease [6]. Within extremity disease, prognostic models have been built largely on clinical and treatment variables; the nomogram of Xue et al., derived from extremity leiomyosarcoma patients in the SEER registry, uses age, tumor grade, size, distant metastasis, and receipt of surgery, and is representative [7]. Vascular invasion has been proposed as an additional histological prognostic marker in soft tissue sarcoma [8,9], and is a component of two established risk classification systems: the SIN system, which combines tumor size, vascular invasion, and microscopic necrosis [10], and the subsequent SING system, which adds peripheral tumor growth pattern [11]. Both were derived from cohorts of mixed histological subtype, and neither has been evaluated in leiomyosarcoma specifically. Vascular invasion is, however, a recommended rather than a core item in the current Royal College of Pathologists dataset for soft tissue sarcoma [12], and is therefore reported less consistently than grade or size. Evidence for its prognostic value in extremity leiomyosarcoma specifically is sparse.

This study examines the prognostic significance of vascular invasion for overall, disease-specific, local recurrence-free, and metastasis-free survival in a single-center cohort of extremity leiomyosarcoma, alongside established clinicopathological factors. The cohort is small and the analysis is intended as exploratory and hypothesis-generating.

## 2. Materials and Methods

### 2.1. Study Design and Setting

This was a retrospective cohort study of consecutive adults treated for extremity leiomyosarcoma at Oxford University Hospitals NHS Foundation Trust. Surgeries were performed between January 2015 and February 2025, with April 2026 serving as the censoring date for all time-to-event analyses. Patients were identified from electronic patient records and the hospital database. The study was registered and approved as a quality improvement project (No. 10674).

### 2.2. Eligibility Criteria

Adults aged 18 years or older were eligible if they had a histopathologically confirmed diagnosis of leiomyosarcoma arising in an extremity, had undergone surgical resection (limb-sparing surgery or amputation) with or without perioperative radiotherapy, had pre-treatment imaging available (extremity MRI and whole-body PET), and had sufficient clinical data for outcome analysis. Patients were excluded if they had a sarcoma other than leiomyosarcoma, leiomyosarcoma of non-extremity origin, recurrent rather than primary disease, insufficient records, inadequate postoperative follow-up at our center, or did not undergo definitive resection of the primary tumor.

**Figure 1:**
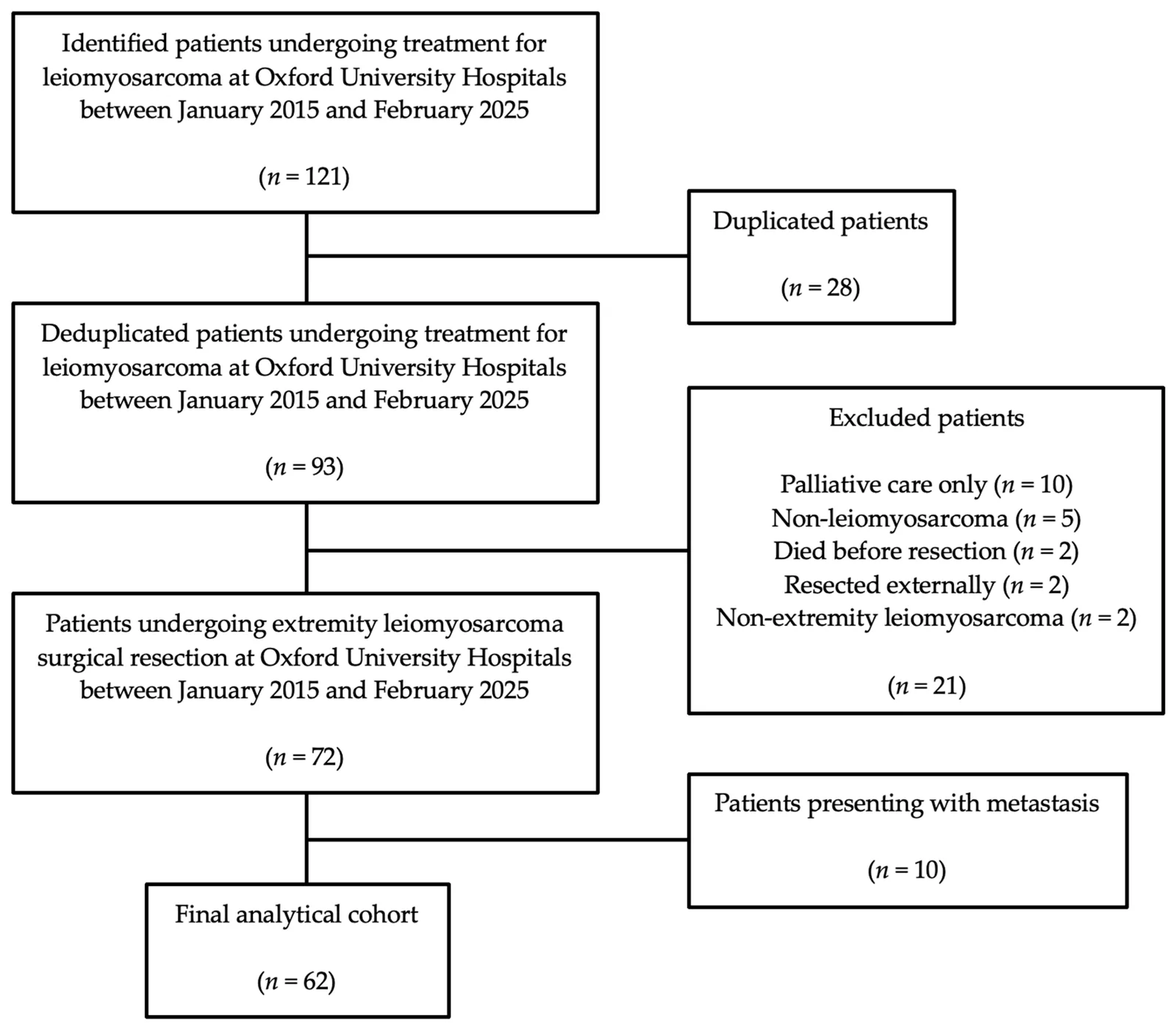
Patient selection flowchart.

The database search returned 121 records. Removal of 28 duplicate records left 93 unique patients, of whom 21 met one or more exclusion criteria, giving 72 patients who underwent resection. Ten of these presented with metastatic disease and underwent resection for local control and symptom management rather than with curative intent; they were excluded, giving a final cohort of 62 patients with localized disease (Figure 1).

### 2.3. Data Collection

Clinicopathological data were extracted from electronic patient records. Variables collected included patient demographics (age, sex, and body mass index), tumor characteristics (anatomical location, laterality, maximum diameter on preoperative MRI, diameter on post-radiotherapy MRI, depth relative to the deep fascia, and FNCLCC histological grade), treatment details (surgical procedure, margin status, neoadjuvant and adjuvant radiotherapy, and systemic therapy) and oncological outcomes (local recurrence, distant metastasis, and vital status at last follow-up).

### 2.4. Histopathological Assessment

**Figure 2:**
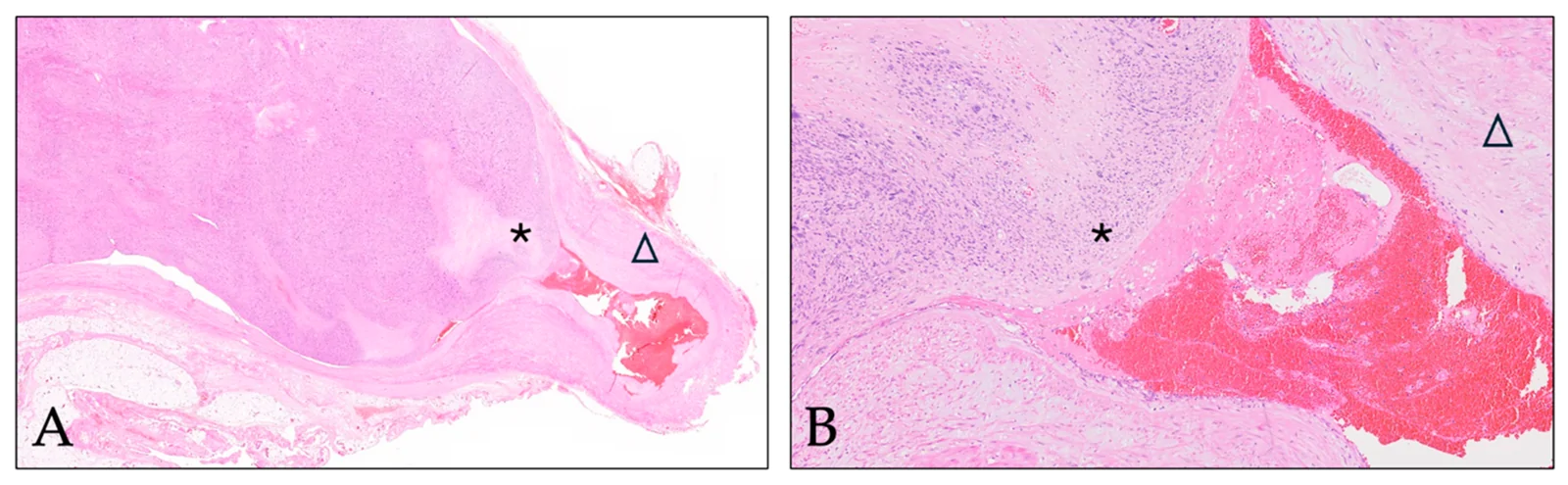
Representative photomicrographs illustrating high-grade extremity leiomyosarcoma with intravascular invasion. (**A**) Low-power and (**B**) higher-power hematoxylin and eosin-stained sections show malignant smooth muscle cells (*) forming a tumor thrombus within a vessel lumen. The triangle (△) denotes the vessel wall.

All specimens were reported by multiple specialist consultant sarcoma histopathologists working within the Oxford Sarcoma Service; cases initially diagnosed at referring institutions were reviewed on referral. Diagnoses were made according to the World Health Organization Classification of *Soft Tissue and Bone Tumours*, fifth edition [13], requiring the presence of characteristic morphological features and appropriate expression of at least two immunohistochemical markers of smooth muscle differentiation. These markers included desmin, smooth muscle actin, caldesmon, muscle specific actin (HHF35), and calponin. Histological grade was assigned using the three-tier FNCLCC system, based on summation of independent scores for differentiation, mitotic index, and amount of necrosis [14]. It was routine practice at our center to record vascular invasion at the time of original diagnostic reporting, based on guidelines from both the Royal College of Pathologists and the College of American Pathologists. Resection specimens were first assessed macroscopically and then sampled for microscopic analysis, typically with one tissue block taken per 10 mm tumor, with additional sampling focused on areas of likely vascular invasion. The presence of vascular invasion was assessed morphologically, or in more challenging cases with the aid of immunohistochemical markers, which highlight endothelial cells, typically ERG and CD31. True vascular invasion was defined by a breach of the endothelial layer indicated by discontinuity in ERG and CD31 staining. Tumor that infiltrated or distorted a vessel wall without breaching the endothelium, including extension within the wall of a vessel from which the tumor arose, was not regarded as vascular invasion. These data were extracted for this study from the original reports; slides were not re-reviewed. A representative example of intravascular invasion in a tumor from this cohort is shown in Figure 2.

### 2.5. Multidisciplinary Management

All patients were discussed at a weekly specialist sarcoma multidisciplinary team meeting attended by orthopedic oncology surgeons, medical and clinical oncologists, histopathologists, radiologists, and sarcoma clinical nurse specialists. Patients referred without a prior diagnosis underwent core needle biopsy; open biopsy was performed where this was non-diagnostic. Staging was performed with whole-body PET and tumors were classified according to the AJCC eighth edition staging system [15].

All 62 patients underwent resection of the primary tumor with curative intent. The decision to deliver radiotherapy in the neoadjuvant or adjuvant setting was made by the clinical oncologist in the context of multidisciplinary discussion; this center predominantly employs neoadjuvant radiotherapy, with adjuvant radiotherapy reserved for selected cases after resection.

### 2.6. Follow-Up Protocol

Following completion of treatment, patients were reviewed at the sarcoma outpatient clinic according to a standardized institutional schedule: quarterly for the first two years, six-monthly for a further three years, and annually thereafter. Each visit included contrast-enhanced MRI of the tumor site and CT of the thorax.

### 2.7. Survival Endpoints and Risk Sets

Four endpoints were defined, each measured from the date of resection. Overall survival (OS) was the time to death from any cause; disease-specific survival (DSS) the time to death attributable to leiomyosarcoma; local recurrence-free survival (LRFS) the time to first biopsy-confirmed local recurrence; and metastasis-free survival (MFS) the time to development of distant metastasis. Patients not experiencing the relevant event were censored at the date of last clinical contact.

The risk set for each endpoint was defined once and applied to all tables and figures. OS and DSS were analyzed in all 62 patients; MFS in 61, with metastasis status not having been recorded in one; and LRFS in 60, with local recurrence status not having been recorded in two.

### 2.8. Statistical Analysis

Continuous variables are summarized as median with interquartile range (IQR) and categorical variables as frequency with percentage. Percentages are calculated against the number of patients with the relevant variable recorded. Survival was estimated by the Kaplan–Meier method with log-transformed confidence intervals, and groups were compared using the log-rank test. Median follow-up was estimated by the reverse Kaplan–Meier method. As only three patients had Grade 1 disease, histological grade was analyzed as a binary contrast of Grade 3 against Grades 1 and 2 combined in both log-rank testing and Cox models.

Vascular invasion, the exposure of interest, was entered into all multivariable models together with FNCLCC grade, an established prognostic factor in this disease. Grade was selected from the factors associated with outcome on log-rank testing, and was the only one associated with all three modeled endpoints (Table A2). Restriction to patients with localized disease removes metastatic status at presentation from the models, in which it would otherwise act as a mediator rather than a confounder. The number of covariates was capped by the requirement to retain at least five events per variable, so tumor size category and tumor depth were not entered into the primary models; both were instead carried into the sensitivity analyses. Kaplan–Meier and log-rank analyses used the maximal risk set available for each endpoint. All Cox models were fitted to patients with complete data on every covariate in the corresponding multivariable model (*n* = 60 for OS and DSS, *n* = 59 for MFS), so that unadjusted and adjusted estimates are directly comparable; grade was not used as a filter for any Kaplan–Meier or log-rank analysis. Baseline characteristics were compared between exposure groups using the Mann–Whitney U test for continuous variables and Fisher’s exact test for categorical variables. The proportional hazards assumption was assessed using scaled Schoenfeld residuals [16].

The number of events per variable was below the conventional threshold of ten for all three models [17]. All multivariable models were therefore refitted using Firth’s penalized partial likelihood [18,19] with profile-likelihood confidence intervals. Model discrimination was quantified by Harrell’s concordance index and corrected for optimism using 1000 bootstrap resamples [20]. Because the covariates were chosen with reference to the log-rank results, the screening step was repeated within each resample so that the correction reflects the whole model-building process; apparent and corrected values are both reported. Optimism-corrected discrimination describes the performance of the fitted model and is not evidence that the association between vascular invasion and outcome is free of confounding. Four sensitivity analyses were performed, each adding a single covariate to the primary model: tumor size category, tumor depth, patient age per ten-year increment, and receipt of neoadjuvant radiotherapy. Six patients died of causes other than leiomyosarcoma, so a sensitivity analysis treated these deaths as a competing event for both disease-specific death and distant metastasis. Cumulative incidence was estimated using the Aalen–Johansen method and compared according to vascular invasion reporting status using Gray’s test. Cause-specific models were retained as the primary analysis, since the aim was to determine whether vascular invasion was associated with the rate of each event among patients who remained at risk. Analyses were performed in R version 4.6.0 (R Foundation for Statistical Computing, Vienna, Austria). A two-sided *p*-value below 0.05 was considered statistically significant.

## 3. Results

### 3.1. Patient and Tumor Characteristics

Sixty-two patients met the eligibility criteria. The median age was 75.5 years (IQR 65.0–81.0) and 38 patients (61.3%) were aged 70 years or older. Thirty patients (48.4%) were male. Median body mass index was 28.0 kg/m^2^ (IQR 23.0–32.0). The lower limb was involved in 52 patients (83.9%), most commonly the thigh (28 patients, 45.2%).

Median maximum tumor diameter on preoperative MRI was 5.0 cm (IQR 3.8–8.2); 32 tumors (52.5%) measured 5 cm or less, 19 (31.1%) measured 5.1–10 cm and 10 (16.4%) exceeded 10 cm. Forty tumors (64.5%) were deep to the deep fascia. Of the 60 patients with an assigned FNCLCC grade, 31 (51.7%) were Grade 3, 26 (43.3%) Grade 2, and three (5.0%) Grade 1. Full characteristics are given in Table 1. Vascular invasion was reported in 11 patients (17.7%) and its reported prevalence did not differ appreciably by tumor size category (15.6%, 10.5%, and 40.0% for tumors of 5 cm or less, 5.1–10 cm and above 10 cm; *p* = 0.152) or by tumor depth (9.1% against 22.5%; *p* = 0.300) but was higher in Grade 3 than in Grade 1–2 tumors (29.0% against 6.9%; *p* = 0.043) (Table A1).

### 3.2. Treatment

Limb salvage surgery was performed in 58 patients (93.5%) and amputation in four (6.5%). An R0 resection was achieved in 58 of the 61 patients with margin status recorded (95.1%), with R1 margins in two (3.3%) and R2 margins in one (1.6%). The median closest margin was 0.2 cm (IQR 0.1–0.3), recorded in 40 patients. Neoadjuvant radiotherapy was administered to 23 patients (37.1%) and adjuvant radiotherapy to seven (11.3%).

### 3.3. Survival Outcomes

Median follow-up, estimated by the reverse Kaplan–Meier method, was 59.5 months (95% CI 42.4–75.5). Twenty-one patients remained at risk at 60 months. Seventeen deaths from any cause occurred, of which 11 were attributed to leiomyosarcoma, and 14 patients developed metastatic disease. Of the 60 patients with a documented final status, 37 (61.7%) were alive with no evidence of disease, six (10.0%) were alive with disease, 11 (18.3%) had died with disease, and six (10.0%) had died of other causes, with two further patients censored at loss to follow-up.

**Figure 3:**
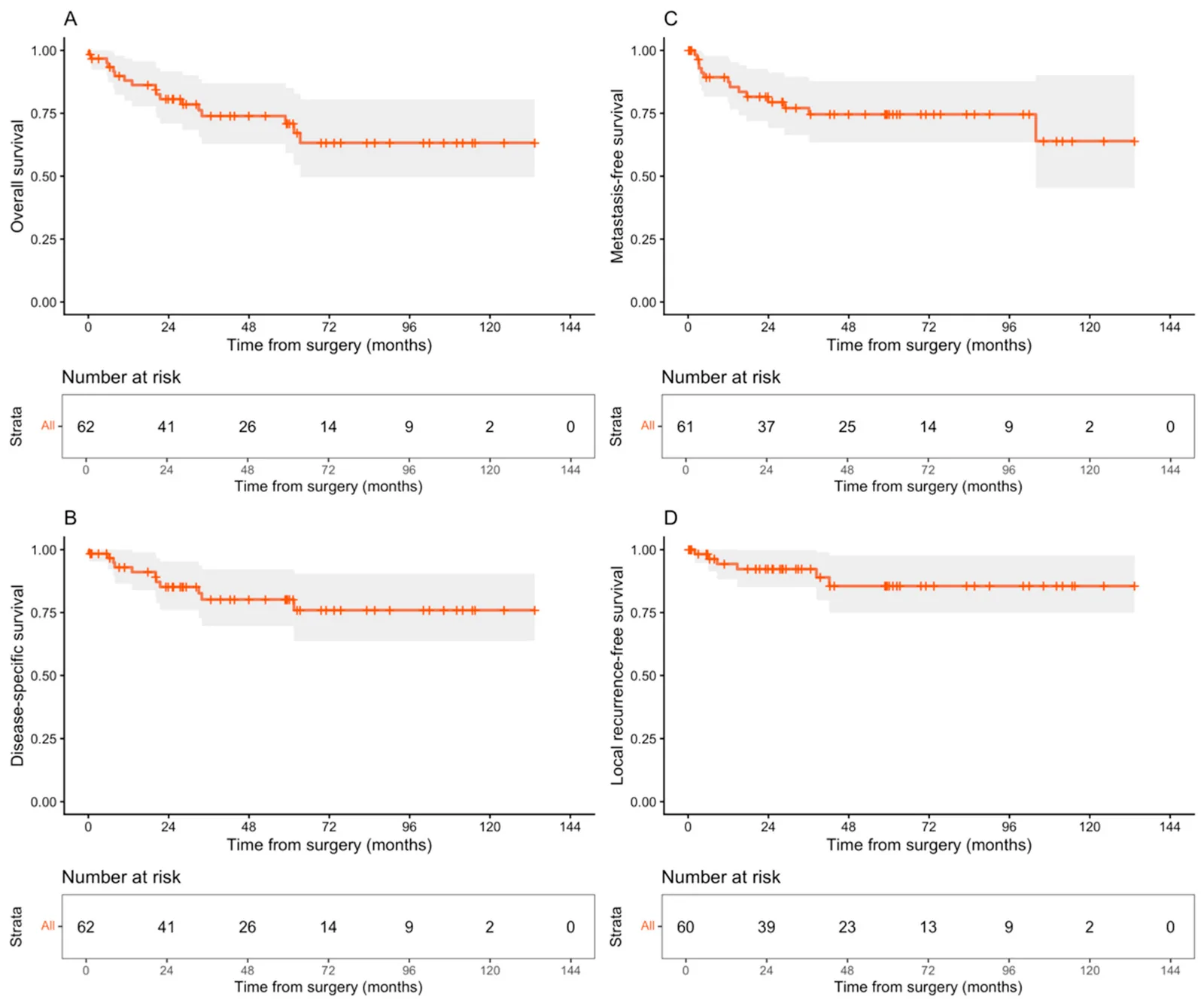
Kaplan–Meier curves for the whole cohort showing overall (**A**), disease-specific (**B**), metastasis-free (**C**), and local recurrence-free survival (**D**), with 95% confidence bands and numbers at risk.

Overall survival was 88.0% (95% CI 80.1–96.8) at one year, 73.9% (95% CI 62.9–86.9) at three years, and 71.0% (95% CI 59.2–85.0) at five years. Corresponding disease-specific survival was 93.0%, 80.2%, and 80.2%; metastasis-free survival was 87.4%, 77.1%, and 74.6%; and local recurrence-free survival was 94.3%, 92.3%, and 85.6%. Estimates with confidence intervals and numbers at risk are given in Table 2 and survival curves are provided in Figure 3. With death from another cause treated as a competing event, the cumulative incidence of death from leiomyosarcoma was 6.8%, 18.8%, and 18.8% at 12, 36, and 60 months, against Kaplan–Meier complements of 7.0%, 19.8%, and 19.8%; for distant metastasis, the corresponding figures were 12.3%, 22.0%, and 24.2% against 12.6%, 22.9%, and 25.4% (Table A5). Competing mortality therefore altered the absolute probabilities by no more than 1.1 percentage points at any time point.

### 3.4. Pattern of Metastasis

Fourteen patients developed metastatic disease during follow-up. The lung was the most frequent site, involved in 13 patients, one of whom also had liver involvement; one patient developed metastasis in the femoral neck.

### 3.5. Radiotherapy

Twenty-three patients received neoadjuvant radiotherapy at a median dose of 50 Gy in 25 fractions, of whom 22 had paired pre- and post-treatment MRI. Median pre-treatment diameter was 5.9 cm (IQR 4.7–8.9) and median post-treatment diameter was 7.2 cm (IQR 4.9–11.7), indicating a median absolute increase of 0.4 cm (IQR 0.0–1.7) and median relative change of 10.8% (IQR 0.0–20.8). Fifteen tumors (68.2%) remained stable in size, defined as an increase below 20% or a decrease below 30%, and seven (31.8%) increased by 20% or more. Vascular invasion was reported in four of the 23 patients who received neoadjuvant radiotherapy (17.4%) and in seven of the 39 who did not (17.9%); Fisher’s exact *p* = 1.000 (Table A1).

Seven patients received adjuvant radiotherapy at a median dose of 60 Gy in 30 fractions. No local recurrence occurred in this subgroup and one patient developed distant metastases.

### 3.6. Univariable Analysis

**Figure 4:**
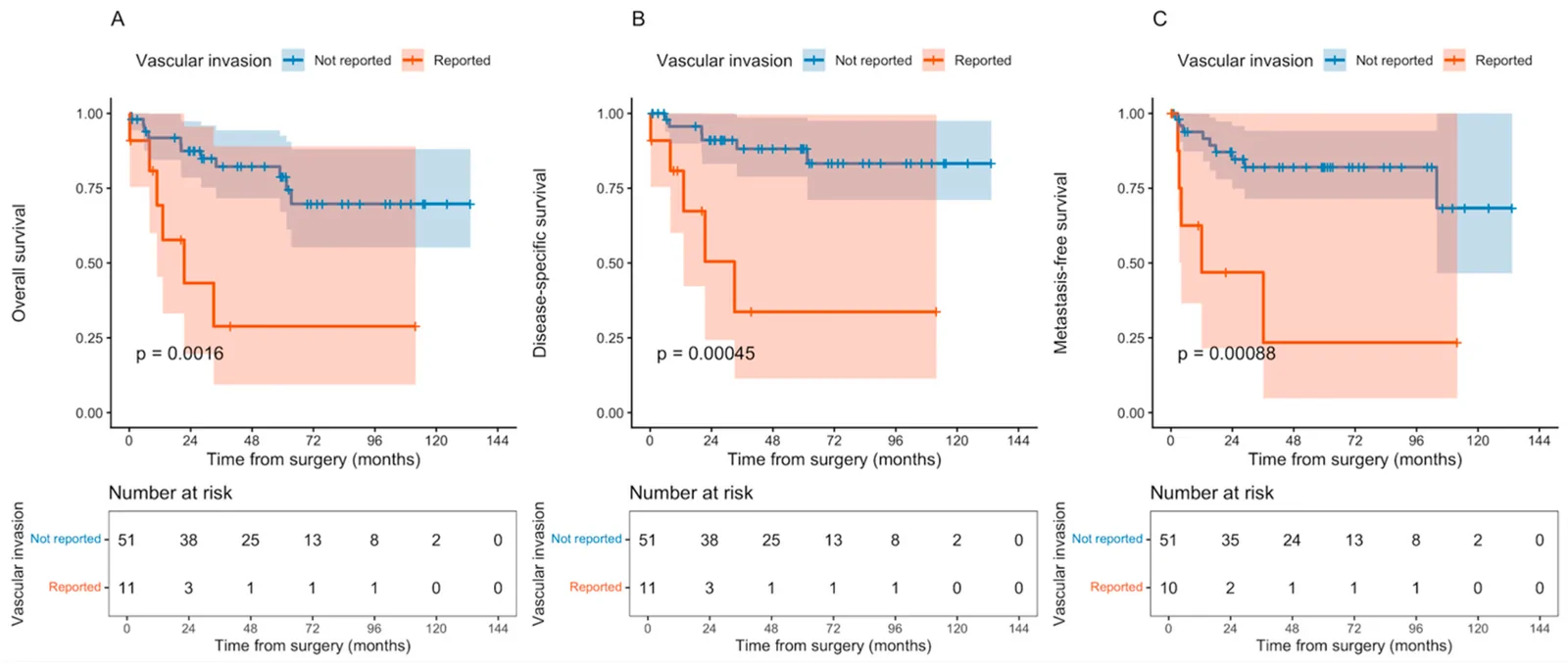
Kaplan–Meier curves for overall (**A**), disease-specific (**B**), and metastasis-free survival (**C**) stratified by vascular invasion reporting status, with 95% confidence bands, log-rank *p*-values, and numbers at risk.

On log-rank testing within the localized cohort, vascular invasion was associated with inferior overall survival (*p* = 0.002), disease-specific survival (*p* < 0.001), and metastasis-free survival (*p* < 0.001), but not with local recurrence-free survival (*p* = 0.631). FNCLCC grade was associated with all four endpoints and tumor size category was associated with overall and disease-specific survival. Tumor depth, margin status, and receipt of neoadjuvant radiotherapy were not associated with any endpoint. Full results are given in Table A2 and curves stratified by vascular invasion are provided in Figure 4. Accounting for competing mortality, the cumulative incidence of death from leiomyosarcoma at 60 months was 59.6%, where vascular invasion was reported against 11.3% where it was not (Gray’s test *p* = 0.002), and that of distant metastasis was 68.8% against 17.3% (*p* = 0.005).

On univariable Cox regression within the same complete-case samples, vascular invasion was associated with inferior overall survival (HR 4.35, 95% CI 1.57–12.10; *p* = 0.005), disease-specific survival (HR 6.42, 95% CI 1.90–21.68; *p* = 0.003), and metastasis-free survival (HR 5.18, 95% CI 1.71–15.72; *p* = 0.004). Unadjusted estimates are shown alongside adjusted estimates in Table 3.

### 3.7. Multivariable Analysis

After adjustment for FNCLCC grade, vascular invasion was associated with inferior overall survival (HR 3.89, 95% CI 1.35–11.26; *p* = 0.012), disease-specific survival (HR 5.48, 95% CI 1.46–20.58; *p* = 0.012), and metastasis-free survival (HR 5.63, 95% CI 1.69–18.80; *p* = 0.005). FNCLCC Grade 3 was associated with inferior disease-specific survival (HR 11.81, 95% CI 1.48–94.11; *p* = 0.020) and metastasis-free survival (HR 8.89, 95% CI 1.95–40.61; *p* = 0.005), and with overall survival at borderline significance (HR 2.85, 95% CI 0.98–8.26; *p* = 0.054) (Table 3).

### 3.8. Model Diagnostics

The number of events per variable was 8.5 for overall survival, 5.5 for disease-specific survival, and 7.0 for metastasis-free survival, which is below the conventional threshold of ten in each case. Refitting the multivariable models using Firth’s penalized partial likelihood gave hazard ratios for vascular invasion of 3.99 (95% CI 1.35–10.96) for overall survival, 5.43 (95% CI 1.49–20.02) for disease-specific survival, and 5.74 (95% CI 1.71–18.24) for metastasis-free survival, with each marginally larger than the corresponding unpenalized estimate. The penalized estimate for FNCLCC Grade 3 in the disease-specific model was 8.13 (95% CI 1.86–76.18) compared with 11.81 (95% CI 1.48–94.11) unpenalized, confirming that the unpenalized value is inflated by sparse data and should not be read as a magnitude.

Apparent concordance indices were 0.706, 0.831, and 0.805 respectively; after bootstrap correction for optimism, with the selection step repeated within each resample, these were 0.667, 0.809, and 0.774 (Table A3). These values describe the discrimination of the fitted models and are not an external validation of the association. No violation of the proportional hazards assumption was detected for any endpoint on global testing (*p* = 0.29, 0.23, and 0.37 for overall, disease-specific, and metastasis-free survival, respectively), and there was no evidence of a time-varying effect of vascular invasion in any model (*p* ≥ 0.29). Using an untransformed time scale, the residuals for FNCLCC grade suggested a possible time-varying effect for overall and disease-specific survival, which is consistent with grade predicting early rather than late events; this did not affect the estimates for vascular invasion (Table A4).

### 3.9. Sensitivity Analyses

Adding tumor size category to the primary model gave a hazard ratio for vascular invasion of 3.93 (95% CI 1.26–12.21; *p* = 0.018) for overall survival, 4.69 (95% CI 1.21–18.24; *p* = 0.026) for disease-specific survival, and 6.92 (95% CI 1.86–25.76; *p* = 0.004) for metastasis-free survival. Adding tumor depth gave 3.38 (95% CI 1.13–10.15; *p* = 0.030), 4.52 (95% CI 1.15–17.73; *p* = 0.031), and 4.60 (95% CI 1.32–16.06; *p* = 0.017), respectively. Adding receipt of neoadjuvant radiotherapy left the estimates essentially unchanged, at 3.96 (95% CI 1.36–11.52; *p* = 0.011), 5.47 (95% CI 1.45–20.57; *p* = 0.012), and 6.57 (95% CI 1.83–23.59; *p* = 0.004); neoadjuvant radiotherapy itself was not associated with any endpoint (*p* = 0.671, 0.883 and 0.392) and there was no interaction between it and vascular invasion (*p* = 0.478, 0.112 and 0.662). Patients with vascular invasion were older than those without (median 83.0 versus 73.0 years; *p* = 0.006); adjustment for age per ten-year increment left the disease-specific and metastasis-free estimates essentially unchanged, at 4.64 (95% CI 1.13–19.05; *p* = 0.033) and 5.85 (95% CI 1.52–22.49; *p* = 0.010), but attenuated the overall survival estimate to borderline significance, at 3.10 (95% CI 0.99–9.71; *p* = 0.053). Estimates for vascular invasion under all five specifications are summarized in Table 4. The disease-specific models rest on 11 events and are underpowered.

## 4. Discussion

In this cohort of 62 patients with localized extremity leiomyosarcoma, vascular invasion was associated with inferior overall, disease-specific and metastasis-free survival after adjustment for histological grade. The pattern of treatment failure is informative. Local control was excellent, with local recurrence-free survival of 94.3% at one year and 85.6% at five years, yet just under a quarter of patients developed distant metastases, and systemic disease accounted for most deaths. Vascular invasion was not associated with local recurrence, although with six local recurrences that analysis is inconclusive and cannot exclude an association. It is worth noting that histological grade did separate local recurrence-free survival on those same events (*p* = 0.010) where vascular invasion did not (*p* = 0.631), which is consistent with vascular invasion marking hematogenous dissemination rather than local aggressiveness without establishing it.

Our survival estimates are consistent with the published literature. Among 1528 patients with extremity leiomyosarcoma in a registry series, five-year overall and cancer-specific survival results were 61.7% and 70.0% [7], against 71.0% and 80.2% here. A systematic review of 13 studies comprising 1380 patients with extremity and trunk wall disease identified tumor size above 5 cm (OR 3.39) and positive margins (OR 2.12) as the dominant adverse factors [21], and the larger institutional series have consistently implicated grade, size, depth, and metastatic status at presentation [5,6,22,23,24]. None of these analyses evaluated vascular invasion. The factors that currently drive risk stratification in extremity leiomyosarcoma are therefore those that are routinely and reliably recorded, which are not necessarily those carrying the most prognostic information.

Vascular invasion is well established as a determinant of metastatic risk. Gustafson et al. combined tumor size above 8 cm, vascular invasion, and microscopic necrosis into the SIN system, classifying tumors with two or three of these features as high risk; five-year metastasis-free survival was 80–85% in the low-risk group against 43–48% in the high-risk group, and the system gave more clinically useful information than AJCC staging [10]. Engellau et al., examining whole-tumor sections from 140 sarcomas, identified vascular invasion in 36% of tumors and found it to be the single strongest predictor of metastasis (HR 3.5); grade, size, and depth carried no independent prognostic value in that model [9]. The subsequent SING model, derived from whole-tumor sections of 239 extremity sarcomas, incorporated vascular invasion alongside size, necrosis, and growth pattern, with each factor carrying a hazard ratio of 2.2 to 2.6 for metastasis [11]. In leiomyosarcoma specifically, intravascular invasion has been reported as an independent predictor of disease-related death [4]. Our point estimates are larger than these, though with wide confidence intervals due to a small sample size, and the direction of association is consistent across all four series.

Leiomyosarcomas of somatic soft tissue are malignant tumors showing smooth muscle differentiation and may arise from the smooth muscle of vessel walls [25]. A tumor whose cell of origin is the tunica media is anatomically positioned to breach the vessel lumen, and direct luminal invasion with formation of tumor thrombus offers access to the systemic circulation that does not depend on the stepwise acquisition of an invasive phenotype. This is consistent with the observation that the association reported here fell on distant metastasis rather than on local recurrence, and with the historical association between intravascular sarcoma thrombus and early systemic spread [26].

Vascular invasion was reported in 17.7% of our tumors, against 36% in the whole-tumor-section series of Engellau et al. [9]. This difference most likely reflects ascertainment rather than biology. Where vascular invasion is reported during routine diagnostic reporting rather than sought systematically, focal involvement will sometimes go unrecorded, and detection scales with the number of blocks examined. Our prevalence estimate should therefore be regarded as a lower bound. Misclassification of this kind would ordinarily be expected to dilute an association rather than create one, and because vascular invasion was reported at original reporting and before any outcome was known, its ascertainment cannot have been influenced by the subsequent clinical course. That argument does not hold automatically where detection is more likely in larger tumors, which also carry a worse prognosis, so we examined it directly: reported prevalence differed little across size categories (15.6%, 10.5%, and 40.0% for tumors of 5 cm or less, 5.1–10 cm and above 10 cm; *p* = 0.152) or by tumor depth (9.1% against 22.5%; *p* = 0.300), and adding tumor size to the models strengthened rather than weakened the association for metastasis-free survival. Vascular invasion was, however, reported more often in Grade 3 than in Grade 1–2 tumors (29.0% against 6.9%; *p* = 0.043), and grade is adjusted for in every model reported here. When the SIN system was tested for reproducibility across two institutions, independent observers agreed on the presence or absence of vascular invasion in 78% of 200 tumors [10], indicating that the feature is recognized consistently once it is sought.

Vascular invasion was reported from the resection specimen, and 23 patients (37.1%) received neoadjuvant radiotherapy, which alters vascular architecture, necrosis, and fibrosis and could affect detectability. Reported prevalence was nonetheless similar in irradiated and non-irradiated specimens (17.4% against 17.9%; *p* = 1.000). Despite irradiated tumors being larger and therefore more extensively sampled, adjusting for neoadjuvant radiotherapy left the estimates for vascular invasion unchanged, and there was no interaction with any endpoint. With four vascular invasion-positive patients among 23 irradiated specimens, however, a difference in detectability cannot be excluded.

Vascular invasion remained associated with each endpoint on separate adjustment for tumor size, tumor depth, and neoadjuvant radiotherapy, with the single exception being overall survival adjusted for age. Size and vascular invasion are not independent, as larger tumors are both more likely to invade vessels and more extensively sampled, and with 17 deaths and 11 disease-specific deaths this cohort cannot reliably separate them; nor does it permit simultaneous adjustment for all established prognostic factors, which is why each was added singly. The consistency of direction across specifications is more informative than any individual estimate.

The tools now used to select patients for perioperative chemotherapy do not include vascular invasion. Sarculator is built on age, tumor size, FNCLCC grade, and histological subtype [27], and PERSARC on age, sex, tumor size, histological subtype, tumor depth, and FNCLCC grade [28]. Given the association reported here and in the Scandinavian studies, this omission may matter. The omission is partly self-perpetuating, as vascular invasion is a recommended rather than a core reporting item [12]. Thus, it is inconsistently reported in the large datasets from which such models are built and cannot be evaluated within them. The immediate implication is not that practice should change on the basis of a single-center series, but that vascular invasion should be documented explicitly, as present or absent, in every extremity sarcoma report, so that its role can be tested at scale.

This study has several limitations. It is a retrospective and single-center study, and the primary analysis rests on 17 deaths, 11 disease-specific deaths, and 14 metastatic events among 62 patients, with only 11 of them reported as having vascular invasion. Events per variable fall below the conventional threshold of ten for every model. Penalized estimation was used to guard against sparse-data bias and the estimates for vascular invasion proved stable under it, but penalization cannot compensate for limited information or for residual confounding, and bootstrap correction of the concordance index describes model discrimination rather than validating the association. The confidence intervals around histological grade remain wide and should not be read as magnitudes. Tumor size and depth could be examined only one at a time, so independence from the full set of established prognostic factors is not established. Vascular invasion status was taken from routine diagnostic reports rather than from dedicated slide review. The Royal College of Pathologists dataset directs that vascular invasion be commented upon where present [12], so positive cases are unlikely to have been missed systematically; but because documentation of absence is not mandated, an absent comment cannot be equated with confirmed absence. Only seven patients received adjuvant radiotherapy, precluding meaningful assessment of that treatment, and MRI-assessed size change after neoadjuvant radiotherapy may reflect treatment-related necrosis, hemorrhage, or edema rather than true tumor response. With six local recurrences, the local recurrence-free survival analysis is uninformative, and its negative result should not be read as evidence that vascular invasion is unrelated to local failure.

## 5. Conclusions

In: this exploratory single-center analysis of 62 patients with localized extremity leiomyosarcoma, vascular invasion reported at routine diagnostic reporting was associated with inferior overall, disease-specific, and metastasis-free survival after adjustment for histological grade, and the association persisted on separate adjustment for tumor size, tumor depth, and neoadjuvant radiotherapy. The number of events is small so the finding is hypothesis-generating rather than a demonstration of a validated independent prognostic factor. It nonetheless supports the routine and explicit documentation of vascular invasion, including its absence, in the histopathological reporting of extremity leiomyosarcoma, so that its prognostic role can be assessed at scale. Prospective multicenter validation with protocolized assessment is required before vascular invasion is used to direct adjuvant therapy.

## Figures and Tables

**Table 1:** Clinicopathological characteristics, treatment, and outcomes.

Characteristic	Value	Denominator
Demographics		
Age at surgery, years—median (IQR)	75.5 (65.0–81.0)	62
Male	30 (48.4)	62
Female	32 (51.6)	62
BMI, kg/m^2^—median (IQR)	28.0 (23.0–32.0)	61
Tumor characteristics		
Lower limb	52 (83.9)	62
Upper limb	10 (16.1)	62
Deep to fascia	40 (64.5)	62
Superficial to fascia	22 (35.5)	62
Maximum diameter, cm—median (IQR)	5.0 (3.8–8.2)	61
FNCLCC Grade 1	3 (5.0)	60
FNCLCC Grade 2	26 (43.3)	60
FNCLCC Grade 3	31 (51.7)	60
Vascular invasion	11 (17.7)	62
Treatment		
Limb salvage surgery	58 (93.5)	62
Amputation	4 (6.5)	62
R0 margin	58 (95.1)	61
R1 margin	2 (3.3)	61
R2 margin	1 (1.6)	61
Neoadjuvant radiotherapy	23 (37.1)	62
Adjuvant radiotherapy	7 (11.3)	62
Outcomes		
Local recurrence	6 (10.0)	60
Distant metastasis	14 (23.0)	61
No evidence of disease	37 (61.7)	60
Alive with disease	6 (10.0)	60
Died of disease	11 (18.3)	60
Died of other cause	6 (10.0)	60
Lost to follow-up	2 (3.2)	62

Percentages are calculated against the denominator given in the final column.

**Table 2:** Kaplan–Meier survival estimates at 12, 36, and 60 months.

Endpoint	Months	At Risk	Events	Survival (%)	95% CI
Overall survival	12	49	7	88.0	80.1–96.8
	36	32	7	73.9	62.9–86.9
	60	21	1	71.0	59.2–85.0
Disease-specific survival	12	49	4	93.0	86.6–99.9
	36	32	6	80.2	69.7–92.2
	60	21	0	80.2	69.7–92.2
Local recurrence-free survival	12	46	3	94.3	88.3–100.0
	36	30	1	92.3	85.3–99.9
	60	19	2	85.6	75.0–97.6
Metastasis-free survival	12	45	7	87.4	79.1–96.6
	36	31	5	77.1	66.4–89.5
	60	20	1	74.6	63.4–87.8

Events are those occurring within each interval.

**Table 3:** Univariable and multivariable Cox proportional hazards models.

Endpoint	Variable	*n*	Events	Unadjusted HR (95% CI)	*p*	Adjusted HR (95% CI)	*p*
OS	Vascular invasion	60	17	4.35 (1.57–12.10)	0.005	3.89 (1.35–11.26)	0.012
	FNCLCC Grade 3	60	17	3.19 (1.12–9.09)	0.030	2.85 (0.98–8.26)	0.054
DSS	Vascular invasion	60	11	6.42 (1.90–21.68)	0.003	5.48 (1.46–20.58)	0.012
	FNCLCC Grade 3	60	11	13.39 (1.71–104.83)	0.013	11.81 (1.48–94.11)	0.020
MFS	Vascular invasion	59	14	5.18 (1.71–15.72)	0.004	5.63 (1.69–18.80)	0.005
	FNCLCC Grade 3	59	14	8.68 (1.93–39.08)	0.005	8.89 (1.95–40.61)	0.005

Both sets of estimates are derived from the same complete-case sample for each endpoint, so that unadjusted and adjusted hazard ratios are directly comparable. OS, overall survival; DSS, disease-specific survival; and MFS, metastasis-free survival.

**Table 4:** Hazard ratios for vascular invasion under five covariate specifications.

Endpoint	Primary Model	Size Added	Depth Added	Age Added	Neoadjuvant RT Added
OS	3.89 (1.35–11.26) *p* = 0.012	3.93 (1.26–12.21) *p* = 0.018	3.38 (1.13–10.15) *p* = 0.030	3.10 (0.99–9.71) *p* = 0.053	3.96 (1.36–11.52) *p* = 0.011
DSS	5.48 (1.46–20.58) *p* = 0.012	4.69 (1.21–18.24) *p* = 0.026	4.52 (1.15–17.73) *p* = 0.031	4.64 (1.13–19.05) *p* = 0.033	5.47 (1.45–20.57) *p* = 0.012
MFS	5.63 (1.69–18.80) *p* = 0.005	6.92 (1.86–25.76) *p* = 0.004	4.60 (1.32–16.06) *p* = 0.017	5.85 (1.52–22.49) *p* = 0.010	6.57 (1.83–23.59) *p* = 0.004

The primary model comprises vascular invasion and FNCLCC grade. Each sensitivity analysis adds a single further covariate to that model. Age is modeled per ten-year increment.

## Data Availability

Due to the sensitive nature of our dataset, the raw data supporting the conclusions of this article will be made available by the authors upon reasonable request.

## Abbreviations

The following abbreviations are used in this manuscript:

AJCC	American Joint Committee on Cancer
BMI	Body mass index
CI	Confidence interval
CT	Computed tomography
DSS	Disease-specific survival
FNCLCC	Fédération Nationale des Centres de Lutte Contre le Cancer
HR	Hazard ratio
IQR	Interquartile range
LRFS	Local recurrence-free survival
MFS	Metastasis-free survival
MRI	Magnetic resonance imaging
OR	Odds ratio
OS	Overall survival
PERSARC	Personalized Sarcoma Care
PET	Positron emission tomography
SEER	Surveillance, epidemiology, and end results
SIN	Size, invasion, necrosis
SING	Size, invasion, necrosis, growth pattern

## Appendix A

**Table A1:** Reported prevalence of vascular invasion by clinicopathological factor.

Factor	Stratum	n	Vascular Invasion Reported, n (%)	Fisher *p*
Neoadjuvant radiotherapy	Not given	39	7 (17.9)	1.000
	Given	23	4 (17.4)	
Tumor size category	5 cm or less	32	5 (15.6)	0.152
	5.1–10 cm	19	2 (10.5)	
	Above 10 cm	10	4 (40.0)	
FNCLCC grade	Grade 1–2	29	2 (6.9)	0.043
	Grade 3	31	9 (29.0)	
Tumor depth	Superficial	22	2 (9.1)	0.300
	Deep	40	9 (22.5)	

*p*-values are from Fisher’s exact test and are given once per factor. These analyses examine whether the reporting of vascular invasion varied with factors that could affect its detection, in particular the extent of tissue sampling and the receipt of neoadjuvant radiotherapy.

**Table A2:** Univariable log-rank analysis.

Endpoint	Variable	n	Log-Rank *p*
Overall survival	Vascular invasion (reported vs. not reported)	62	0.002
	FNCLCC Grade 3 (vs. Grade 1–2)	60	0.022
	Tumor size category	61	0.001
	Deep (vs. superficial)	62	0.051
	Margin status (R0 vs. R1/R2)	61	0.976
	Neoadjuvant radiotherapy (vs. none)	62	0.397
Disease-specific survival	Vascular invasion (reported vs. not reported)	62	<0.001
	FNCLCC Grade 3 (vs. Grade 1–2)	60	0.001
	Tumor size category	61	0.003
	Deep (vs. superficial)	62	0.128
	Margin status (R0 vs. R1/R2)	61	0.671
	Neoadjuvant radiotherapy (vs. none)	62	0.584
Local recurrence-free survival	Vascular invasion (reported vs. not reported)	60	0.631
	FNCLCC Grade 3 (vs. Grade 1–2)	58	0.010
	Tumor size category	59	0.249
	Deep (vs. superficial)	60	0.155
	Margin status (R0 vs. R1/R2)	59	0.323
	Neoadjuvant radiotherapy (vs. none)	60	0.276
Metastasis-free survival	Vascular invasion (reported vs. not reported)	61	<0.001
	FNCLCC Grade 3 (vs. Grade 1–2)	59	<0.001
	Tumor size category	60	0.101
	Deep (vs. superficial)	61	0.101
	Margin status (R0 vs. R1/R2)	60	0.356
	Neoadjuvant radiotherapy (vs. none)	61	0.274

Analyses use the maximal risk set available for each endpoint and variable.

**Table A3:** Model diagnostics and internal validation.

Model	Events	Covariates	EPV	Penalized VI HR (95% CI)	Penalized Grade 3 HR (95% CI)	Apparent c	Corrected c	Optimism
OS	17	2	8.5	3.99 (1.35–10.96)	2.68 (1.00–8.11)	0.706	0.667	0.038
DSS	11	2	5.5	5.43 (1.49–20.02)	8.13 (1.86–76.18)	0.831	0.809	0.022
MFS	14	2	7.0	5.74 (1.71–18.24)	7.26 (2.13–37.65)	0.805	0.774	0.031

Firth-penalized estimates are shown for both covariates, together with model discrimination before and after bootstrap correction for optimism (1000 resamples). The correction repeats the covariate selection step within every resample so that it reflects the whole model-building process. Corrected discrimination describes the performance of the fitted model and is not an external validation of the association. VI, vascular invasion; EPV, events per variable; and c, Harrell’s concordance index.

**Table A4:** Assessment of the proportional hazards assumption using scaled Schoenfeld residuals.

Model	Term	Chi-Square	df	*p*	*p* (Untransformed Time)
OS	Vascular invasion	0.30	1	0.587	0.963
	FNCLCC Grade 3	2.30	1	0.130	0.034
	Global test	2.48	2	0.289	0.098
DSS	Vascular invasion	1.09	1	0.296	0.528
	FNCLCC Grade 3	2.05	1	0.152	0.030
	Global test	2.92	2	0.233	0.094
MFS	Vascular invasion	0.41	1	0.521	0.743
	FNCLCC Grade 3	1.90	1	0.168	0.621
	Global test	1.99	2	0.371	0.881

*p*-values are derived from the Grambsch–Therneau test. The primary analysis uses a Kaplan–Meier transformation of the time scale; results using untransformed time are given for comparison. OS, overall survival; DSS, disease-specific survival; and MFS, metastasis-free survival.

**Table A5:** Cumulative incidence of death from leiomyosarcoma and of distant metastasis, accounting for competing mortality.

Endpoint	Months	1—Kaplan–Meier (%)	Cumulative Incidence (%)	Difference (%)	Competing Event (%)
Death from leiomyosarcoma	12	7.0	6.8	0.2	5.1
	36	19.8	18.8	1.0	7.3
	60	19.8	18.8	1.0	10.2
Distant metastasis	12	12.6	12.3	0.3	5.3
	36	22.9	22.0	0.9	7.4
	60	25.4	24.2	1.1	10.2

Cumulative incidence is estimated by the Aalen–Johansen estimator with death from a cause other than leiomyosarcoma as the competing event. The final column gives the cumulative incidence of the competing event itself. Estimates for distant metastasis use the 61 patients with metastasis status recorded.
